# 劳力型热射病致弥散性血管内凝血1例报告并文献复习

**DOI:** 10.3760/cma.j.cn121090-20241225-00588

**Published:** 2024-12

**Authors:** 莉 张, 朝辉 张, 中毅 孙, 小云 徐, 高生 周

**Affiliations:** 1 武汉大学中南医院重症医学科，武汉 430071 Department of Critical Care Medicine, Zhongnan Hospital of Wuhan University, The Second Clinical College of Wuhan University, Wuhan 430071, China; 2 三峡大学第一临床医学院（宜昌市中心人民医院）重症医学科，宜昌 443003 Department of Critical Care Medicine, The First College of Clinical Medical Science, China Three Gorges University; Yichang Central People's Hospital, Yichang 443003, China; 3 宜昌市脓毒症临床医学研究中心，宜昌 443003 Yichang Clinical Medical Research Center for Sepsis, Yichang 443003, China

**Keywords:** 中暑, 凝血功能, 弥散性血管内凝血, 抗凝, Heat stroke, Coagulation function, Disseminated intravascular coagulation, Anticoagulation

## Abstract

**目的:**

探讨劳力型热射病导致弥散性血管内凝血（DIC）发生的机制及临床表现。

**方法:**

通过对宜昌市中心人民医院收治的1例劳力型热射病患者的临床数据进行分析，结合相关文献进行复习，评价热射病与DIC之间的关联，并总结临床表现和实验室检查结果。

**结果:**

患者在高温环境下进行剧烈劳动后，出现高热、意识障碍等症状。实验室检查显示PLT 43×10^9^/L、D-二聚体5.3 mg/L、凝血酶原时间21.8 s，符合DIC诊断。经过及时的降温补液，合理的抗凝、目标导向的替代治疗及支持治疗后，患者逐渐好转。

**结论:**

劳力型热射病可导致DIC的发生，其机制可能与高温引起的全身炎症反应、血管内皮损伤及血流动力学改变有关。早期识别DIC的风险并采取综合治疗措施，对于改善患者的预后具有重要意义。

## Abstract (en)

**Objective:**

To explore the mechanisms and clinical manifestations of disseminated intravascular coagulation（DIC）caused by exertional heat stroke.

**Methods:**

Analyze the clinical data of a patient with exertional heat stroke in Yichang Central People's Hospital and review relevant literature to evaluate the association between heat stroke and DIC, summarizing clinical presentations and laboratory findings.

**Results:**

The patient exhibited symptoms such as high fever and altered consciousness after engaging in intense labor in a high-temperature environment. Laboratory tests showed a platelet count of 43×10^9^/L, D-dimer level of 5.3 mg/L, and prothrombin time of 21.8 s, which are consistent with a diagnosis of DIC. Following prompt cooling, fluid resuscitation, appropriate anticoagulant, goal-directed replacement therapy and supportive therapy, the patient's condition gradually improved.

**Conclusion:**

Exertional heat stroke can lead to DIC, and clinical attention should be paid to its early recognition and intervention. Effective coagulation management and timely anticoagulant and supportive therapy are crucial for improving prognosis.

劳力型热射病（exertional heat stroke，EHS）是一种由高温环境和剧烈劳动导致的急性热应激反应，表现为高热、意识障碍和多脏器功能损害等症状。随着全球气候变化及户外劳动强度的增加，EHS发生率逐渐上升。EHS疾病进展迅速，致死率可达20％～50％[1]。因此，及时识别和有效干预成为临床工作的重点。热射病患者常伴随凝血功能异常，表现为凝血酶原时间（PT）延长、D-二聚体升高等，这与热应激引发的内源性炎症反应密切相关[1]。弥散性血管内凝血（DIC）是严重的凝血障碍，EHS发生时其发病率达11.2％[2]。DIC是由多种诱因引起的血液凝血和纤溶系统的失衡，导致微血管内大量血栓形成，并对机体微循环造成损害。研究[3]显示，EHS可通过促炎因子的释放诱导DIC的发生，进而增加患者的死亡风险。因此，及时识别和管理DIC对于改善EHS患者的预后至关重要。本文探讨1例EHS合并DIC的患者，分析其临床特征、实验室检查结果及治疗过程，并对近年来相关文献进行回顾，全面总结EHS和DIC之间的相互关系及其临床影响。

## 病例资料

患者男，56岁，建筑工人，因“被人发现意识丧失9 h”于2021年8月1日送至宜昌市中心人民医院。患者当天外出作业（实时温度38 °C），约3 h后被人发现倒地，意识丧失，呼之不应，皮肤温度高、干燥，左下肢皮肤烫伤，小便失禁，紧急送至当地医院。测腋下体温41 °C，急诊抢救室立即予以冰毯、风扇物理降温，4 °C生理盐水静脉输注，左下肢皮肤消毒包扎，完善颅脑CT未见明显异常，考虑诊断为“中暑”。因患者持续高热，并出现严重腹泻，排大量褐色稀便，伴血压进行性下降，家属要求转至我院治疗，急诊以EHS收入急诊重症监护病房。既往体健，无特殊疾病史、冶游史、家族遗传病史。入院时查体：肛温40.3 °C、脉率147次/min、呼吸频率35次/分、血压92/51 mmHg（1 mmHg = 0.133 kPa，去甲肾上腺素1.5 µg·kg^−1^·min^−1^）、指端脉搏血氧饱和度93％（面罩吸氧8 L/min）；神志模糊、球结膜充血、皮肤穿刺点瘀斑、导尿管见少量酱油色尿液、胃肠减压管可见咖啡色液体；双肺可闻及湿性啰音、心律齐、肠鸣音活跃、双下肢无水肿。左下肢皮肤烫伤10％体表面积（total body surface area，TBSA），Ⅱ度烧伤，腐皮剥脱，创面基底红白相间。

完善辅助检查：动脉血气分析示pH为7.23、二氧化碳分压（PCO_2_）27.3 mmHg、氧分压（PO_2_）128.7 mmHg、实际碳酸氢盐（HCO_3_^-^）17.81mmol/L、乳酸9.47 mmol/L；血常规示WBC 3.31×10^9^/L、HGB 158.00 g/L、PLT 43×10^9^/L；凝血功能示PT 21.8s、纤维蛋白原（Fib）1.01g/L、活化部分凝血活酶时间（APTT）51.7 s、凝血酶时间（TT）19.2 s、D-二聚体5.3 mg/L、血栓弹力图CI值为-16.2；生化分析示血钾3.05 mmol/L、尿素氮（BUN）8.62 mmol/L、肌酐（Cr）211 µmol/L、谷丙转氨酶（ALT）103 U/L、谷草转氨酶（AST）648 U/L、白蛋白23.52 g/L、LDH 1 234 IU/L、肌酸激酶（CK）25 240 IU/L、肌酸激酶同工酶（CK-MB）480 IU/L、肌钙蛋白I 0.047 ng/ml、B型钠尿肽（BNP）147 ng/L、肌红蛋白>900 ng/ml；大便潜血试验示3+，胃内容物隐血试验示2+。心电图示窦性心动过速。心脏彩超和下肢动静脉超声均未见异常。头颅和胸部CT示双侧基底节区少许腔隙性脑梗死、肺感染。腹部彩超示胆囊结石，肝胰脾、泌尿系未见异常。国际血栓与止血学会弥散性血管内凝血诊断标准（ISTH-DIC）评分（表1）为7分；急性生理与慢性健康评分系统（APACHE Ⅱ）评分为26分。

**表1:** 国际血栓与止血学会弥散性血管内凝血诊断标准评分

指标	0分	1分	2分	3分
PLT（×10^9^/L）	≥100	50～100	<50	–
PT延长值（s）	<3	3~6	≥6	–
纤维蛋白原（g/L）	≥1.0	<1.0	–	–
D-二聚体（µg/ml）	<2.5	–	2.5～5	≥5

**注** PT：凝血酶原时间；总分：≥5分可诊断热射病性弥散性血管内凝血；“–”表示该指标不计分或不作为诊断标准的一部分

入院诊断：EHS；DIC；横纹肌溶解综合征；急性肾损伤；休克；急性心肌损伤；急性肝损伤；消化道出血；低钾血症；左下肢Ⅱ度烧伤（10％ TBSA）；肺感染。

入科后立即给予急诊重症常规护理，气管插管接有创呼吸机辅助通气，冰毯、冰袋持续降温，补液升压维持循环，床旁连续性肾脏替代治疗（CRRT），镇痛镇静降低机体氧耗，甘油果糖防治脑水肿，甲强龙、乌司他丁抗炎，哌拉西林他唑巴坦钠抗感染，泮托拉唑保护胃黏膜，还原性谷胱甘肽护肝，维持水电解质平衡及对症治疗，请烧伤科会诊协助处理左下肢皮肤烫伤。同时结合患者病史、凝血指标和ISTH-DIC评分诊断为DIC，APTT、PT明显延长，Fib、PLT明显降低，血栓弹力图结果提示低凝状态，且有消化道出血症状，给予补充凝血物质（新鲜冰冻血浆800 ml、冷沉淀凝血因子10 u、单采血小板1治疗量），小剂量肝素（1 µg·kg^−1^·h^−1^）抗凝，并每2小时复查凝血功能，根据情况调整肝素剂量并适当补充凝血物质（表2），以维持APTT在基础值的1.5倍左右。

**表2:** 劳力型热射病合并弥散性血管内凝血患者入院第1天至第5天的凝血变化情况

指标	第1天00:00	第1天4:00	第1天8:00	第1天12:00	第1天20:00	第2天8:00	第3天8:00	第4天8:00	第5天8:00
APTT（s）	51.7	88.5	60.1	83.4	77.4	70.5	74.6	69.6	80.4
PT（s）	21.8	26.7	21.1	22.4	18.4	16.6	16.0	14.4	15.3
TT（s）	19.2	23.7	19.2	19.9	20.2	19.1	20.5	17.5	18.1
Fib（g/L）	1.01	0.77	1.10	1.59	1.89	2.01	2.28	3.78	4.55
D-二聚体（mg/L）	5.3	7.4	4.7	4.3	4.1	2.7	1.7	4.7	7.1
FDP（mg/L）	23.4	30.1	19.7	17.9	18.1	9.6	9.2	18.7	21.2
PLT（×10^9^/L）	43	21	26	35	39	61	114	79	39
ISTH-DIC评分（分）	7	8	7	6	5	3	0	3	2

**注** APTT：活化部分凝血活酶时间；PT：凝血酶原时间；TT：凝血酶时间；Fib：纤维蛋白原；FDP：纤维蛋白降解产物；ISTH-DIC：国际血栓与止血学会弥散性血管内凝血诊断标准

入院第3天，患者体温降至正常，凝血功能、内环境逐渐好转。入院第5天，患者再次出现发热，最高体温达38.3°C，伴氧合指数逐渐下降，血压进行性下降需大剂量去甲肾上腺素维持，同时CRRT管路中的动脉壶、静脉壶和滤器均频繁出现血栓形成情况（图1）。第5天复查PT 15.3 s、Fib 4.55 g/L、APTT 80.4 s、TT 18.1 s、D-二聚体7.1 mg/L、纤维蛋白降解产物（FDP）21.2 mg/L、抗凝血酶Ⅲ（AT-Ⅲ）活性44％。动脉血气分析（FiO_2_ 0.7）示pH 7.37、PCO_2_ 46.6 mmHg、PO_2_ 94.1 mmHg、乳酸4.59 mmol/L、氧合指数134.4mmHg；WBC 11.7×10^9^/L、中性粒细胞百分比78.9％、HGB 97.00 g/L、PLT 39×10^9^/L、C反应蛋白184.4 mg/L、降钙素原31.74 ng/ml。给予脉搏指示连续心输出量监测血流动力学，结果提示高排低阻，心输出量指数（PCCI）4.17 L·min^−1^·m^−2^、全心舒张末期容积指数（GEDI）852 ml/m²、系统血管阻力指数（SVRI）1 086 dyn.s.cm-5.m^2^、血管外肺水指数（ELWI）11 ml/kg；血培养示敏感大肠埃希菌感染，肺泡灌洗液病原学检测结果为敏感肺炎克雷伯菌，考虑脓毒血症、脓毒性休克及脓毒症性凝血病。调整抗菌药物为美罗培南（1.0 g，每8小时1次），肺保护性通气参数设置及俯卧位通气改善呼吸情况，同时调整CRRT模式为CVVH前稀释模式串联HA380灌流器清除炎症介质。在凝血方面，下调肝素剂量，辅以枸橼酸钠体外抗凝，同时补充单采血小板维持PLT在50×10^9^/L以上。

**图1:**
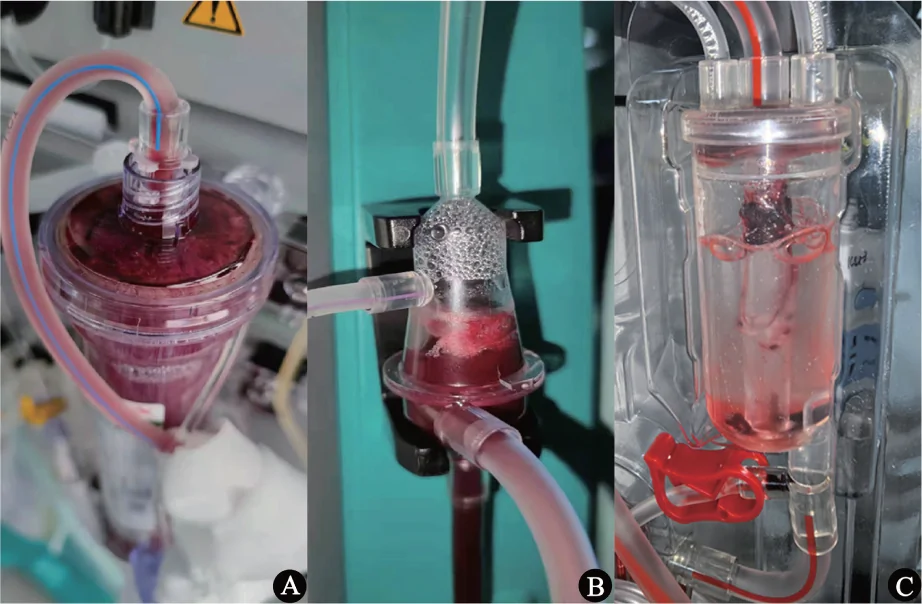
劳力型热射病合并弥散性血管内凝血患者治疗过程中CRRT管路血栓形成 **A** 滤器血栓；**B** 静脉壶血栓；**C** 动脉壶血栓

治疗4天后，患者血压逐渐趋于稳定，去甲肾上腺素减停，体温及炎症指标下降，凝血指标较前好转，尿量逐渐恢复。因此，停用了枸橼酸钠，逐渐上调肝素剂量至目标值（表3）。患者于入院第13天终止血液透析，第14天顺利拔除气管导管，第16天转回当地医院治疗。2周后随访，患者好转出院。

**表3:** 劳力型热射病合并弥散性血管内凝血患者入院第5天至第16天的凝血变化情况

指标	第5天	第6天	第7天	第8天	第9天	第10天	第12天	第14天	第16天
APTT（s）	80.4	59.1	54.2	55.0	67.5	78.9	60.4	65.3	41.1
PT（s）	15.3	13.6	13.7	14.3	15.0	13.3	14.8	12.9	11.7
Fib（g/L）	4.55	3.27	3.30	2.94	3.76	2.01	1.97	2.62	2.33
D-二聚体（mg/L）	7.1	7.2	5.8	5.9	5.5	3.2	2.9	2.1	2.7
FDP（mg/L）	21.2	44.2	29.8	26.5	25.1	14.7	9.9	9.4	6.9
PLT（×10^9^/L）	39	13	27	33	93	121	176	211	267
AT-Ⅲ活性（％）	44	56	54	62	75	79	72	75	81

**注** APTT：活化部分凝血活酶时间；PT：凝血酶原时间；TT：凝血酶时间；Fib：纤维蛋白原；FDP：纤维蛋白降解产物；AT-Ⅲ活性：抗凝血酶Ⅲ活性

## 讨论并文献复习

该患者在高温下进行体力作业后出现高热伴意识丧失，外院诊断为中暑并进行初步降温后转至我院明确诊断为EHS。EHS是一种致命性中暑，其高死亡率可能与热细胞毒性、凝血功能紊乱、肠道继发感染、全身炎症反应综合征及多器官功能衰竭等各种复杂的临床特征相互作用有关[1]。热射病患者常表现为凝血功能的异常，主要表现为PLT和Fib进行性下降、FDP和D-二聚体升高或阳性、PT和APTT明显延长、AT活性下降，及时进行血液学检查和监测对于发现DIC至关重要。本例入院时即对其完善了包含血栓弹力图在内的凝血指标检测，根据2023年《热射病性凝血病诊疗中国专家共识》[4]，推荐应用ISTH-DIC评分系统诊断热射病性DIC，本例DIC评分达到7分，提示其DIC症状较重。在伴随的凝血指标中，患者的PT、APTT显著延长，Fib与PLT较低，D-二聚体显著升高，均证实了DIC的存在。

热射病导致的DIC，是患者多器官功能衰竭，甚至死亡的重要原因之一[5]。11％～48％的热射病患者可发生DIC[4]。在高温环境下，机体通过发汗和血管扩张等机制来调节体温，但在剧烈运动和极端高温条件下，这些机制可能失效，导致体温急剧升高[6]。热射病时导致的炎症反应失调、凝血功能紊乱和微循环障碍是DIC的主要发病机制[3],[7]。在此过程中，肌肉溶解、休克和炎症反应会进一步加重组织缺氧，从而加快DIC的发生。患者在入院时出现的消化道出血和酱油色尿液提示其存在内源性出血和肾脏损伤，而这些症状与DIC的病理生理机制相一致。此外，患者在治疗过程中出现病情变化，血流动力学评估结果显示高排低阻的状态，此时全身循环降低，微血管内血液流动变得缓慢，使得微血栓形成的风险增高。结合该病例的临床表现，肺感染和脓毒血症进一步引发全身炎症反应，促使凝血机制失衡，形成持续的微血管内血栓，是DIC常见的特征。

面对热射病相关DIC，首先依赖于有效的体温控制与综合管理策略，旨在纠正体液和电解质失衡，维持血流动力学稳定、抗炎和改善凝血功能等。近年来，针对体温管理的研究逐渐增多，强调了个体化的温度管理策略在临床中的重要性。采用物理降温、药物干预等多种手段进行综合管理，能够有效降低热射病患者的体温，从而减少并发症的发生率[8]。研究表明，热疗和冷疗的结合应用能够在一定程度上改善患者的预后，提高生存率[9]。除此之外，在严密监测过程中合理进行抗凝和替代治疗尤为重要[10]。然而，抗凝治疗的实施需要根据患者的具体情况进行个体化调整，以平衡出血风险和血栓风险。在无活动性出血的情况下，应对患者尽早启动抗凝[5]，通过抗凝减少凝血物质的过度消耗，进而延缓DIC的进展。目前常用的抗凝药物主要为普通肝素和低分子肝素，其通过使多种凝血因子失活，间接抑制凝血酶激活从而抑制血栓形成[11]。因普通肝素半衰期短、监测便捷，且可用鱼精蛋白中和，临床上使用广泛。但低分子肝素出血风险比普通肝素低，安全性更高[12]。另外，甲磺酸萘莫司他也用于DIC的抗凝治疗[13]–[14]，出血高风险EHS可选用[4]。与此同时，对有出血倾向或发生出血的热射病性凝血病患者，应采用目标导向的替代治疗，主要包括凝血因子、纤维蛋白原及血小板等[4]。通过补充凝血因子和血小板可有效纠正患者凝血功能障碍，减少出血风险。

本例患者通过物理降温、输注大量液体、应用去甲肾上腺素维持循环稳定，保证了其在急救阶段的基本生命体征。在凝血功能方面，给予新鲜冰冻血浆、冷沉淀和单采血小板，能够快速弥补失去的凝血因子及血小板，逐步改善患者凝血紊乱状态。随着患者病情的逐步稳定，对肝素的使用量进行了合理调整，以期维持APTT在合适的范围，避免过度出血和血栓形成的风险。在随后的治疗过程中，患者感染加重，及时调整抗菌药物来控制感染，同时在CRRT治疗中调整模式以清除体内的炎症介质，促进患者的总体康复。在患者血小板严重低下、出血风险高的状态下，适当下调肝素剂量，并辅以枸橼酸钠进行体外抗凝以维持CRRT管路的通畅运行。随后的监测发现，患者的血压逐渐稳定，凝血指标逐渐恢复正常，体温和炎症指标也相继下降，这表明我们采取的治疗方案是有效的。患者最终在经过持续的综合治疗后成功出院，提示在合并热射病和DIC病例的临床管理中需要进行细致的监测和个体化的治疗策略。

综上所述，该患者为少有的救治后好转的重症EHS病例。早期识别DIC的发生并进行了降温、抗凝补凝以及各器官功能支持等综合治疗，在严密监测凝血指标的基础上，精准调整抗凝药物的剂量，最终患者的凝血功能逐渐好转，康复出院。这与文献报道[15]的结果一致，及时的降温及纠正凝血功能可显著改善EHS患者的预后。未来的研究应关注热射病相关DIC的预防策略和早期干预策略，以期为类似病例的管理提供更为有效的指导。
